# Preliminary Transcriptomic Insights into the Combined Pathogenesis of Avian Leukosis Virus and *Salmonella pullorum* Co-Infection

**DOI:** 10.3390/vetsci13030283

**Published:** 2026-03-18

**Authors:** Min Tan, Rong Ran, Cheng Liu, Tao Xie, Keshan Zhang, Qigui Wang, Xi Lan, Haiwei Wang

**Affiliations:** 1 Chongqing Academy of Animal Sciences, Chongqing 402460, China; tanmin123@email.swu.edu.cn (M.T.); zhangks@cqaa.cn (K.Z.); wangqigui@hotmail.com (Q.W.); 2 College of Animal Science, Southwest University, Chongqing 400715, China; wrjrk154927@email.swu.edu.cn (R.R.); a15310313040@email.swu.edu.cn (C.L.); a524448729@email.swu.edu.cn (T.X.); 3 Chongqing Engineering Research Center of Higher Education for Herbivore, Chongqing 400715, China

**Keywords:** avian leukemia, pullorum disease, transcriptome sequencing, co-infection, molecular mechanism

## Abstract

Co-infection with avian leukemia and Pullorum Disease severely compromises poultry health, yet its pathogenic mechanisms remain unclear. We employed transcriptome sequencing to analyze gene expression changes and enriched pathways in kidney, spleen, and liver tissues of Chongqing Chengkou mountain chickens under single-infection (avian leukemia virus or Pullorum Disease) and co-infection conditions. Significant differences were observed in the number and pathways of differentially expressed genes between co-infected and single-infected groups. These genes were predominantly enriched in pathways involving extracellular matrix–receptor interactions, PPAR signaling, and calcium ion signaling. RT-qPCR validation confirmed significant upregulation of MAPK10 and SQLE, alongside downregulation of genes such as FOXG1. This study identifies multiple differentially expressed genes and pathways associated with immunity and tumorigenesis, providing crucial molecular insights into the regulatory mechanisms underlying avian leukemia and Pullorum Disease co-infection.

## Simple Summary

Co-infection with avian leukemia and Pullorum Disease poses a serious threat to poultry health. However, the combined pathogenic mechanism remains unclear, and no commercial vaccines or effective drugs are available. This study used transcriptome sequencing to identify differentially expressed genes and pathways related to immunity and tumorigenesis. The findings provide new insights into the co-infection mechanism and offer a theoretical basis for developing integrated control strategies in the poultry industry.

## 1. Introduction

Avian Leukemia (AL) and Pullorum Disease (PD) are two major infectious diseases that severely threaten the health of poultry in China. These diseases not only cause substantial economic losses to the poultry industry but also threaten the long-term sustainability and economic viability of China’s poultry farming sector [1,2]. AL and PD are caused by the avian leukosis virus (ALV) and *Salmonella pullorum* (*S. pullorum*), respectively. ALV, a member of the retrovirus family, induces immunosuppression, delays multi-organ development, and promotes tumorigenesis in the host, leading to reduced egg production, increased mortality, and higher culling rates [3]; *S. pullorum* is a Gram-negative intracellular bacterium [4] that primarily affects chicks, causing high mortality and reproductive disorders in breeding stock, thereby impairing chicken growth and development [3]. To date, no commercial vaccines or specific antiviral drugs are available for the prevention and control of AL. Although significant progress has been made in vaccine research for pullorum disease—particularly with the development of live [5], attenuated [6,7], and genetically engineered vaccines [8]—the complexity of *S. pullorum* and the influence of environmental factors [9] mean that prevention and control still require integration with biosecurity measures, rational drug use, and the development of novel vaccines and therapeutics to achieve more effective outcomes. Therefore, the most widely recognized and effective approach to controlling AL and PD is to implement purification measures starting from the breeding stock [10]. During recent purification efforts for AL and PD in the core breeding flock of Chengkou Mountain Chickens, we observed that these two diseases frequently co-occur: the infection rate of AL was significantly higher in flocks positive for PD than in those negative for it. Flocks co-infected with both diseases exhibited more severe clinical symptoms and significantly higher mortality rates than those infected with either disease alone. This finding aligns with the results reported by Huang et al. [11], based on testing of breeding flocks across multiple regions nationwide. Consequently, a thorough investigation into the regulatory mechanisms governing the interaction between AL and PD is crucial for the efficient development of integrated control strategies.

Much is known about how ALV and *S. pullorum* infect hosts individually [12,13]. However, their combined pathogenic mechanisms during co-infection are still unclear. Early studies have identified the potential threat of co-infection with ALV and *S. pullorum* to poultry health. Huang Jianqiang et al. [11] first detected co-infection with ALV-J and *S. pullorum* in chicken flocks in Shandong Province and developed relevant animal models. Jing Yuanyuan et al. [14] found that ALV-J infection causes immunosuppression and enhances *S. pullorum* colonization, while *S. pullorum* infection may increase ALV-J viral loads via inflammatory responses. These findings suggest a mutually reinforcing pathogenic relationship between the two pathogens. Recent research has increasingly focused on the immunological mechanisms driving this interaction. Ying Tang et al. [15] observed that *S. pullorum* infection shifts the immune response toward a Th2-dominant profile [16,17], characterized by decreased IFN-γ and increased IL-4 levels [18]. This immune polarization may facilitate persistent infection by these pathogens, highlighting the importance of understanding immune responses in managing co-infections in poultry.

Within the broader field of viral-bacterial co-infection research, evidence has emerged indicating that certain pathogen co-infections form immune–inflammatory interaction networks [19]. Viruses may influence host antibacterial immunity through pathways such as interferon signaling, while bacterial infections can modify the local microenvironment, thereby affecting viral replication. These mechanisms offer theoretical frameworks for understanding the complex interactions among different pathogens. However, in the context of ALV-*S. pullorum* co-infection, systematic genome-wide evidence is still lacking regarding the existence of such mutually reinforcing relationships, the specific molecular mechanisms involved, and how these interactions collectively reshape the host’s transcriptional regulatory networks across multiple organs [20].

Building on these findings, this study performed transcriptome sequencing on target organs from chicken populations infected with ALV alone, *S. pullorum* alone, and ALV-*S. pullorum* co-infection. By comparing immune-related pathways and tumor proliferation pathways across the three groups, we identified key regulatory factors and screened potential candidate genes associated with chicken immunity. These results provide a theoretical basis for further elucidating the molecular mechanisms underlying co-infection with AL and PD, thereby advancing understanding of pathogen interactions and host responses.

## 2. Materials and Methods

### 2.1. Ethical Statement for Laboratory Animals

All animals utilized in this study were obtained from the Chongqing Chengkou Mountain Chicken Genetic Resources Institute. The experimental procedures involving animals strictly complied with the Regulations on Laboratory Animal Management of Southwest University, along with all relevant rules and regulations. The research protocol was thoroughly reviewed and approved by the Animal Experiment Ethics Review Committee of Southwest University, receiving formal approval under the reference number SWU_LAC2025111041. Throughout the entire experimental process, the principles of the 3Rs—reduction, replacement, and refinement—were rigorously followed to ensure the highest standards of animal welfare and ethical research conduct.

### 2.2. Principal Instruments and Reagents

Information on the principal reagents and instruments used in this study is provided in Table 1 and Table 2.

### 2.3. Confirmation and Collection of Samples

#### 2.3.1. Experimental Grouping

To investigate the combined pathogenic mechanism of co-infection by ALV and *S. pullorum*, this study established four experimental groups with clearly defined infection statuses based on serological diagnostic results. The procedure was as follows: First, a preliminary screening of the chicken population at the Chengkou Mountain Chicken Genetic Resource Center was conducted using the *Salmonella pullorum* plate agglutination test. Rectal swabs were collected from individuals testing positive in the agglutination test. Based on kit instructions and preliminary validation, the eluate obtained from these swabs was suitable for further testing. Subsequently, ALV antigen analysis was performed on these eluates using an ALV-p27 antigen ELISA detection kit. Based on the combined results of these tests, chickens were divided into four groups:

Group A (Co-infection group): Agglutination test (+), ALV antigen (+), *n* = 3.

Group B (Single ALV infection): Agglutination test (−), ALV antigen (+), *n* = 3.

Group C (Single *S. pullorum* infection): Agglutination test (+), ALV antigen (−), *n* = 3.

Group D (Healthy control): Agglutination test (−), ALV antigen (−), clinically asymptomatic, *n* = 3.

#### 2.3.2. Sample Size and Experimental Design

The sample size for this study was limited to three chickens per group, primarily due to the practical challenges associated with screening and confirming pure single infections versus co-infections within field flocks. To maintain statistical rigor, each chicken was considered an independent biological replicate. All comparisons between groups and subsequent statistical analyses were performed at this biological replicate level. To gather more comprehensive information on host responses and improve data robustness within the constraints of a limited number of biological replicates, a multi-tissue sampling strategy was implemented. Tissue samples were collected from the liver, spleen, and kidneys of each chicken, resulting in a total of 36 samples for transcriptomic analysis. This approach allowed for a more detailed understanding of tissue-specific responses and increased the reliability of the data despite the small sample size.

#### 2.3.3. Sample Collection

Chickens were anesthetized with intravenous sodium pentobarbital and euthanized by cervical bleeding. After confirming death, the abdominal cavity was incised along the medial thigh. Using sterile, autoclaved instruments, liver, spleen, and kidney tissues were aseptically collected from each chicken. The tissue blocks were rinsed with pre-chilled PBS, then cut into approximately 50 mg pieces and transferred to centrifuge tubes containing RNAlater RNA stabilizer. Following overnight stabilization at room temperature, the stabilizer was discarded, and tissue pieces were stored at −80 °C in an ultra-low temperature freezer.

### 2.4. RNA-Seq Library Preparation and Sequencing

The collected samples were sent to Novogene Bio-Technology Co., Ltd. (Beijing, China) for transcriptome sequencing. Upon receipt, RNA was extracted from the tissue using standard methods, followed by rigorous quality control, library preparation, and sequencing of the RNA samples, ensuring high-quality data for analysis.

### 2.5. Transcriptome Sequencing Data Analysis

Low-quality and contaminated reads were removed from the raw Fastq data using FastQC, based on specific criteria: reads containing adapter sequences, reads with N-base content exceeding 10%, and reads with low-quality bases (Q ≤ 5) accounting for over 50% were discarded, resulting in high-quality Clean Reads. Next, HISAT2 (v2.2.1) was used to align these Clean Reads to the chicken reference genome (GCF_016699485_2). Only reads successfully mapped to the genome were retained for further analysis. Gene expression quantification was performed with featureCounts (v2.0.6), producing raw counts for each gene, which served as input for DESeq2. Differential expression analysis was conducted using the DESeq2 R package (v1.42.0). This software models count data with a negative binomial distribution and normalizes raw counts using the Median of Ratios method to account for sequencing depth differences. Genes with significant differential expression were identified based on a Benjamini–Hochberg-corrected *p*-value (FDR or padj) ≤ 0.05 and an absolute log2(FoldChange) ≥ 1. For these genes, GO functional enrichment and KEGG pathway analyses were performed using the clusterProfiler R package (v4.8.1). The significance threshold for enrichment was set at a corrected *p*-value ≤ 0.05.

### 2.6. Candidate Gene Screening

To validate sequencing results and explore key mechanisms of co-infection, eight genes were selected from differential expression analysis for RT-qPCR validation. The criteria included genes with a corrected *p*-value ≤ 0.05 and |log2FC| ≥ 1. The selected genes also showed high magnitude of expression change and functional relevance to infection-related pathways like immunity, inflammation, or tissue remodeling.

### 2.7. Real-Time Quantitative PCR (RT-qPCR) Analysis

To validate the reliability of transcriptomic sequencing results, five randomly selected differentially expressed genes were analyzed using real-time quantitative PCR (RT-qPCR). Primers were designed for quantitative analysis, and log2 fold-change (log2FC) values were compared with the sequencing data. The qPCR conditions were as follows: 94 °C pre-denaturation for 30 s, 98 °C denaturation for 10 s, annealing at 56.8 °C for 30 s, and extension at 72 °C for 60 s, repeated for 35 cycles. Primer sequences are listed in Table 3.

## 3. Results

### 3.1. Quality Control Analysis of Transcriptome Data

Transcriptome sequencing of 36 samples yielded 239.62 gigabytes of high-quality reads for subsequent analysis, with each sample averaging over six gigabytes of data. All samples produced an average of 44.37 million clean reads, exhibiting an average data validity rate exceeding 96% and an average Q30 of 95.55%. Compared to the chicken reference genome, the average total mapping rate was 90.11%, with a unique mapping rate of 84.90%. Detailed data are presented in Appendix A and Appendix B.

### 3.2. Correlation Analysis Among Samples

**Figure 1:**
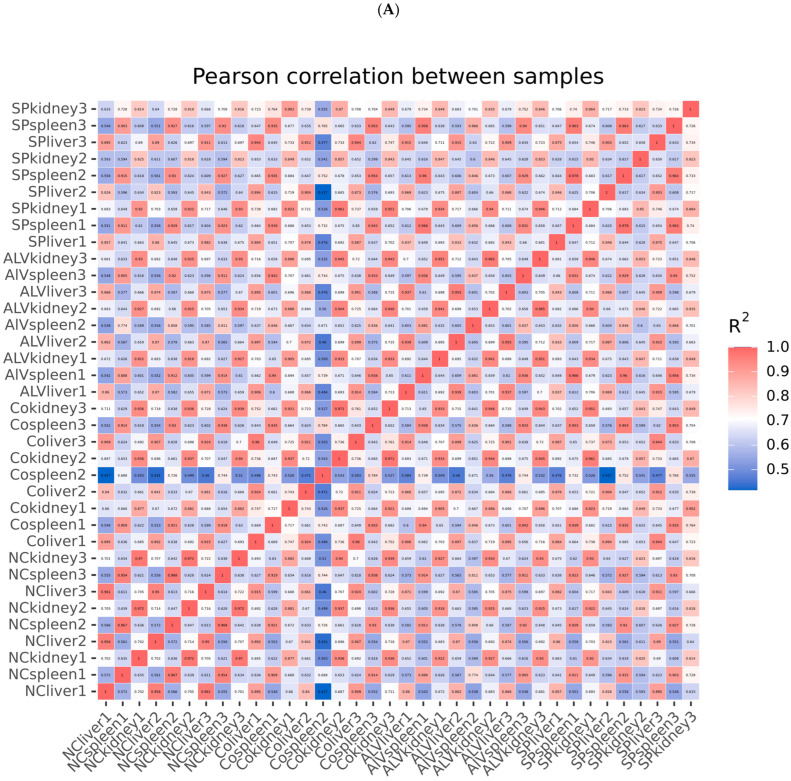

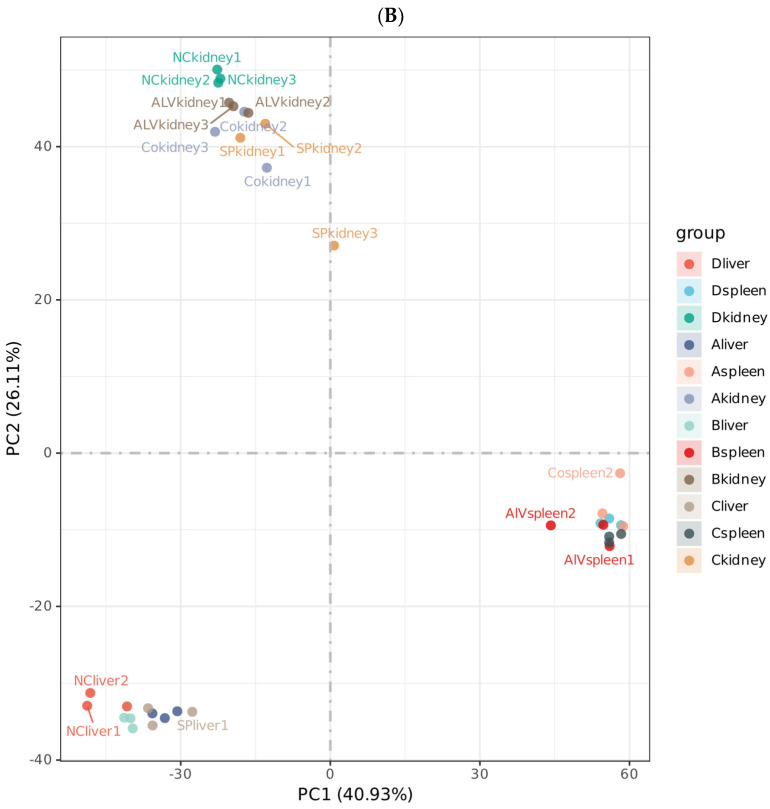
Correlation analysis between samples. Pearson correlation analysis heatmap of different organizational samples (**A**) and principal component analysis of different organizational samples (**B**).

**Figure 2:**
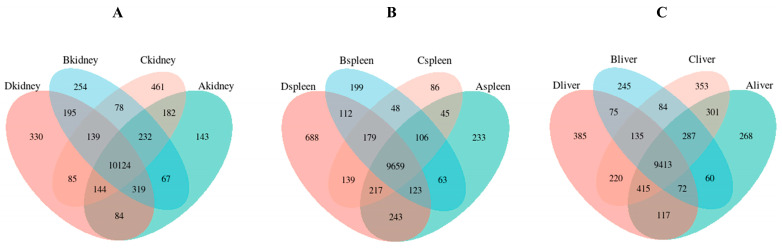
Co-expression Venn analysis of three different treatment samples in kidney (**A**), spleen (**B**) and liver (**C**).

Correlation matrices were constructed for the FPKM distributions of 36 samples using Pearson correlation and principal component analysis (PCA). Co-expression Venn diagrams were plotted for genes across different visceral groups. Results indicate that within the same visceral organ, samples from the same group exhibit high correlation coefficients, similar expression patterns, and good intra-group reproducibility (Figure 1A). Conversely, samples from different visceral organs show distinct distributions along PC1 and PC2 (Figure 1B), demonstrating high consistency in sequencing results across groups and strong reproducibility of sequencing data. Figure 2 clearly illustrates the similarities and differences in gene expression between the three treatment groups across kidney, spleen, and liver tissues: In kidney tissue, Group A shared the fewest co-expressed genes with Group D (only 84), while Group B shared the most (195). In the spleen, Group A and Group D shared the highest number of co-expressed genes (243), while Group B and Group D shared the fewest (112). In the liver, Group C and Group D shared the highest number of co-expressed genes (220), and Group B and Group D shared the fewest (75). These results indicate that the treatment effect of Group A exhibited the greatest differential expression in the kidney, while the treatment effect of Group B showed the greatest differential expression in both the spleen and liver.

### 3.3. Transcriptome Sequencing Differential Gene Expression Analysis

**Figure 3:**
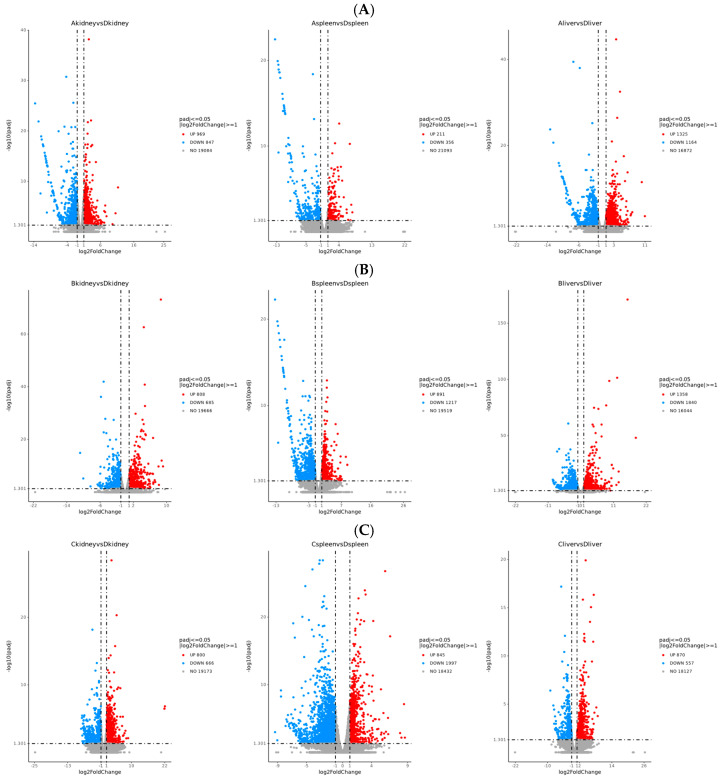
Analysis of differentially expressed genes. Volcano plots of differentially expressed genes (**A**–**C**).

**Figure 4:**
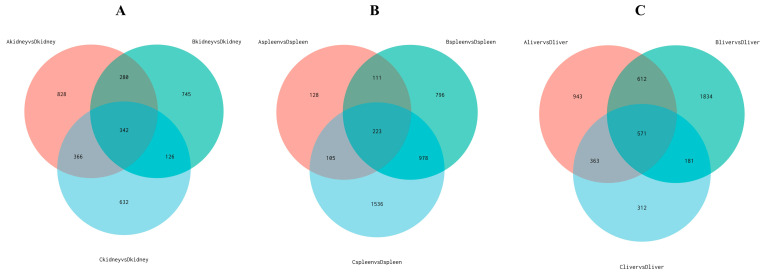
Analysis of differentially expressed genes. (**A**–**C**) Venn diagrams showing differentially expressed genes in the kidney, spleen, and liver, respectively.

**Figure 5:**
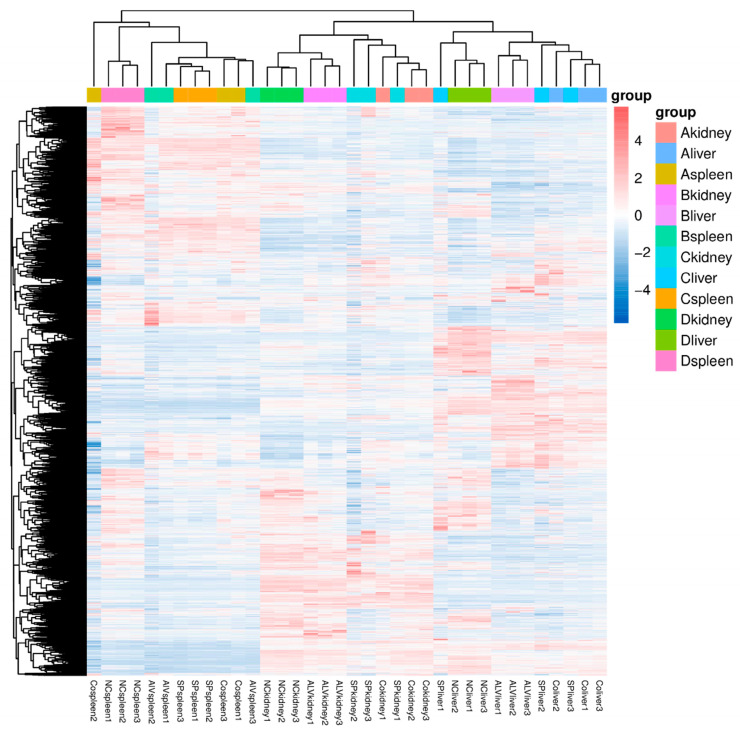
Cluster analysis of differentially expressed genes.

Differential gene expression analysis of kidneys, livers, and spleens from Groups A, B, and C was performed using DESeq2 software. The screening criteria included a corrected *p*-value ≤ 0.05 and an absolute log2 fold-change ≥ 1. In the kidneys, Group A exhibited 1816 differentially expressed genes (DEGs), with 969 upregulated and 847 downregulated. Group B showed 1493 DEGs, comprising 808 upregulated and 685 downregulated genes. Group C had 1466 DEGs, including 800 upregulated and 666 downregulated genes (Figure 3A). In the spleen, Group A displayed 567 DEGs, with 211 upregulated and 356 downregulated. Group B contained 2108 DEGs, with 891 upregulated and 1217 downregulated. Group C had 2842 DEGs, including 845 upregulated and 1997 downregulated genes (Figure 3B). In the liver, Group A showed 2489 DEGs, with 1325 upregulated and 1164 downregulated. Group B had 3198 DEGs, comprising 1358 upregulated and 1840 downregulated. Group C contained 1427 DEGs, with 870 upregulated and 557 downregulated (Figure 3C). A Venn diagram analysis of DEGs (Figure 4) indicated that the combined effect of avian leukemia virus and S. Pullorum infection on chickens was not simply additive across tissues. Co-infection with ALV and S. Pullorum likely induced combined or antagonistic interactions within the host, resulting in novel gene expression patterns distinct from those observed in single infections. Cluster analysis of these differentially expressed genes revealed that samples from the same group clustered together, indicating satisfactory gene clustering results and reasonable grouping within this sample set (Figure 5).

### 3.4. Functional Enrichment Analysis of Differentially Expressed Genes

**Figure 6:**
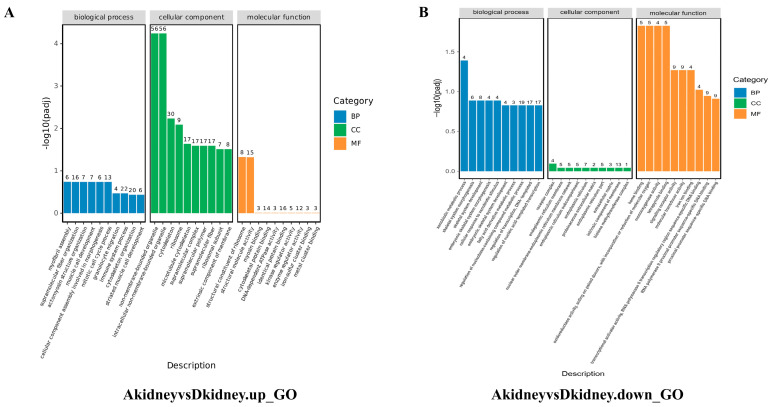
GO enrichment results of significantly differentially expressed genes in the kidney within the mixed-infection group. (**A**) Enrichment analysis of up-regulated genes; (**B**) Enrichment analysis of down-regulated genes.

**Figure 7:**
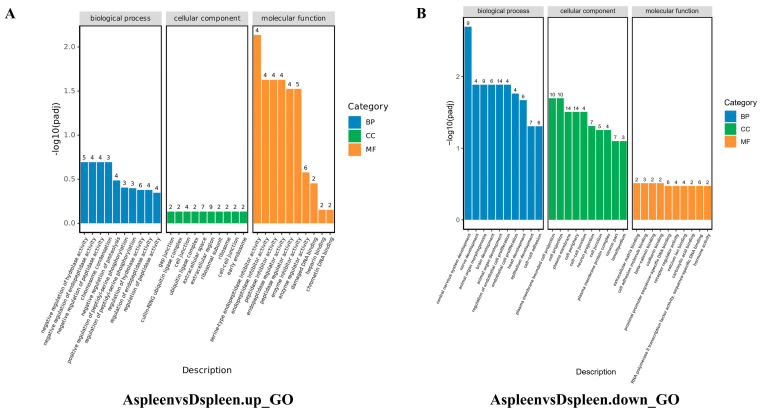
GO enrichment results of significantly differentially expressed genes in the spleen within the mixed-infection group. (**A**) Enrichment analysis of up-regulated genes; (**B**) Enrichment analysis of down-regulated genes.

**Figure 8:**
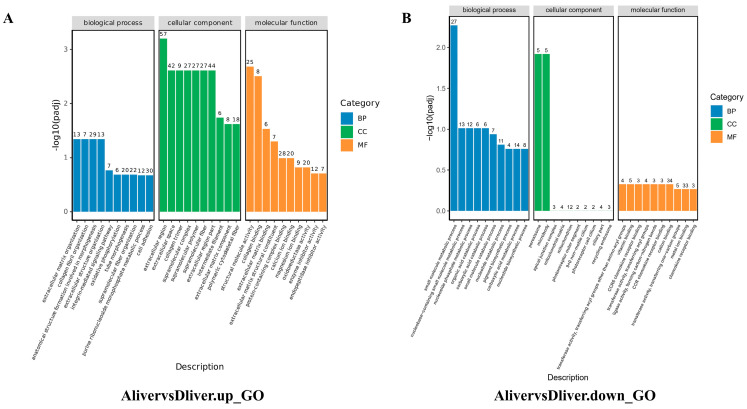
GO enrichment results of significantly differentially expressed genes in the liver within the mixed-infection group. (**A**) Enrichment analysis of up-regulated genes; (**B**) Enrichment analysis of down-regulated genes.

GO functional enrichment analysis of differentially expressed genes yielded results categorized into three major groups: biological process, cellular component, and molecular function. The top 10 most significantly enriched functional categories within each group were selected for analysis. The results revealed that in the kidney (Figure 6), Group A’s upregulated genes were significantly enriched in the ribosome biosynthesis pathway, whilst downregulated genes were concentrated in the metabolism of exogenous substances and redox enzyme activity processes. In the spleen (Figure 7), Group A’s upregulated genes showed significant enrichment in enzyme inhibitor activity, whereas downregulated genes were markedly enriched in the regulation of endothelial cell proliferation and intercellular adhesion. In the liver (Figure 8), upregulated genes were significantly enriched in extracellular matrix processes, whilst downregulated genes showed significant enrichment in small molecule metabolism and peroxisome function. Conversely, the regulatory patterns of Groups B and C diverged from Group A’s multi-organ “synthetic activation and metabolic inhibition” combined regulatory pattern. In the kidney, Group B genes were significantly concentrated in chromosome segregation processes, whilst Group C genes clustered around oxidative phosphorylation. In the spleen and liver, the single-infection group primarily enriched pathways related to basal metabolism, such as cell acylamide metabolism, nucleotide metabolism, and specific cellular functions like ribosomal structure. Detailed data are presented in Appendix C and Appendix D.

**Figure 9:**
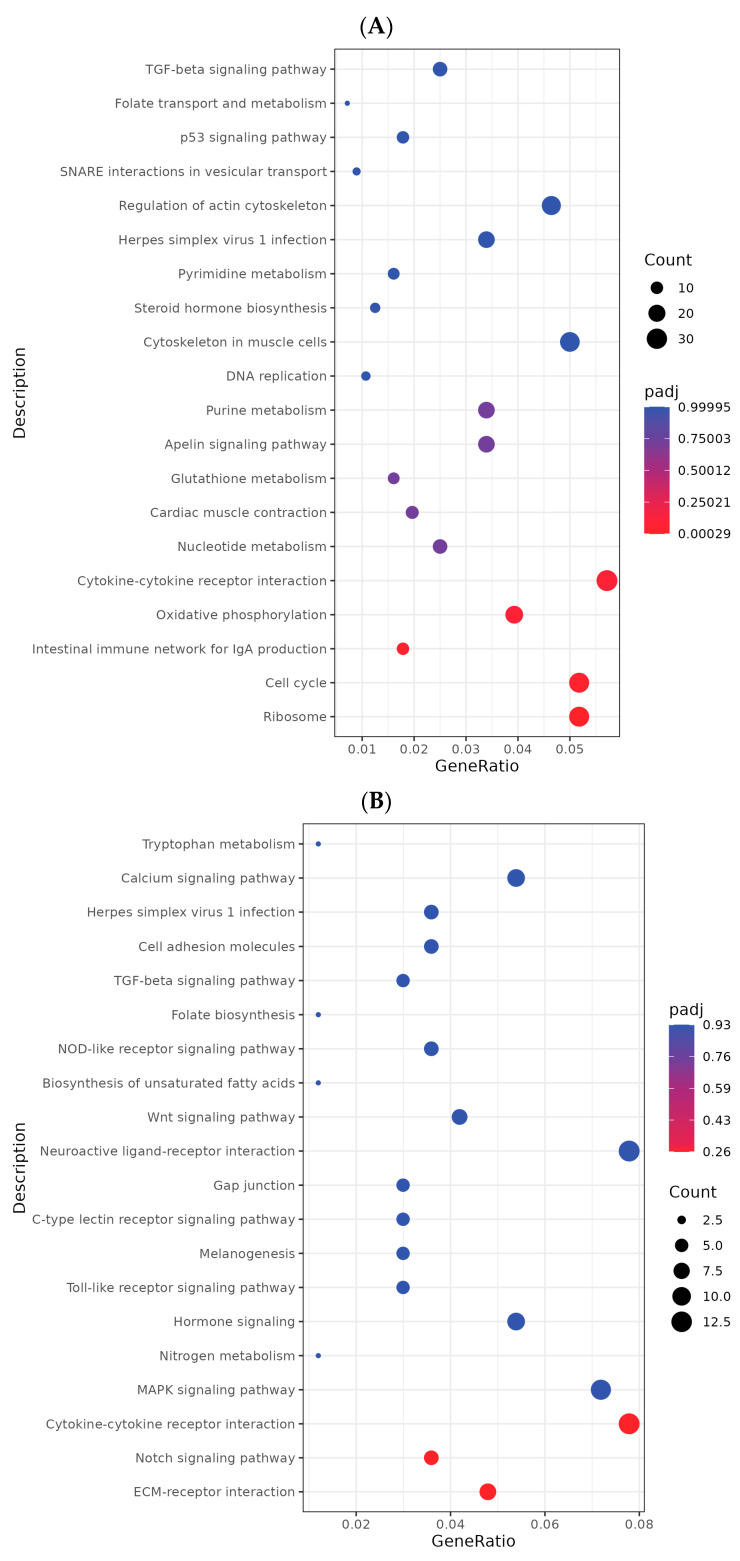

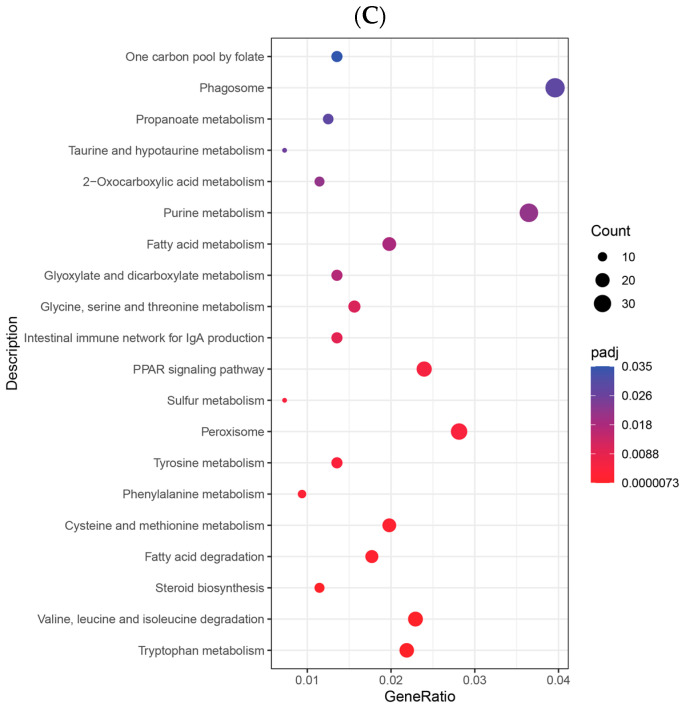
KEGG pathway enrichment analysis of differentially expressed genes. Bar charts (**A**–**C**) respectively present the KEGG enrichment results for differentially expressed genes in the kidneys, spleen, and liver within the co-infection group.

Furthermore, KEGG enrichment analysis revealed the following: in renal tissue, differentially expressed genes in Group A were significantly enriched in three core pathways: immune and inflammatory responses (e.g., cytokine–cytokine receptor interactions), biosynthesis (e.g., ribosomes), and energy metabolism (e.g., oxidative phosphorylation). Conversely, the effects of single-infection groups were more targeted: Group B genes were primarily enriched in metabolic pathways such as steroid biosynthesis; Group C genes were concentrated in oxidative phosphorylation and the transforming growth factor-β signaling pathway. Inter-group differences revealed that compared to Group B, Group A’s differentially expressed genes predominantly enriched in steroid biosynthesis and PPAR signaling pathways; no significantly enriched pathways were observed between Groups A and C. In spleen tissue, no KEGG pathways reached the significance threshold for enrichment in Group A. Conversely, the effects of the single-infection group were pronounced: Group B’s differentially expressed genes were predominantly enriched in metabolic and biosynthetic pathways such as ribosome and oxidative phosphorylation; Group C exhibited widespread impacts on cellular structure and adhesion, with genes significantly enriched in pathways including cell adhesion molecules, focal adhesions, ECM–receptor interactions, and calcium signaling. Differences between Group A and Groups B/C were limited to pathways such as muscle cytoskeleton. In the liver, Group A differentially expressed genes were significantly enriched in a core metabolic regulatory cluster encompassing amino acid metabolism, fatty acid metabolism, peroxisome function, and PPAR signaling pathways (Figure 9). Both Groups B and C showed significant enrichment in numerous metabolism-related pathways, with Group C exhibiting the most extensive enrichment, spanning multiple levels from amino acid degradation to lipid metabolism. Compared to Group B, Group A exhibited more pronounced enrichment of differentially expressed genes in the PPAR signaling pathway and ECM–receptor interactions.

### 3.5. Screening of Candidate Genes

Based on differential gene expression analysis, with screening criteria of an adjusted *p*-value ≤ 0.05 and |log2(FoldChange)| ≥ 1, eight significantly differentially expressed genes were identified, comprising four downregulated genes and four upregulated genes. The specific screening results are as follows: SQLE (upregulated 4.8-fold), MSMO1 (upregulated 4.4-fold), FDFT1 (downregulated 7-fold), FOXG1 (downregulated 5.3-fold), LOC107049046 (downregulated 12-fold), MAPK10 (upregulated 4.8-fold), FN1 (upregulated 2.9-fold), and LOC101747704 (upregulated 5.8-fold). Functional annotation and pathway analysis revealed that these candidate genes were predominantly enriched in pathways including the extracellular matrix–receptor interaction pathway and the intercellular adhesion pathway.

### 3.6. RT-qPCR Validation of Differentially Expressed Genes

**Figure 10:**
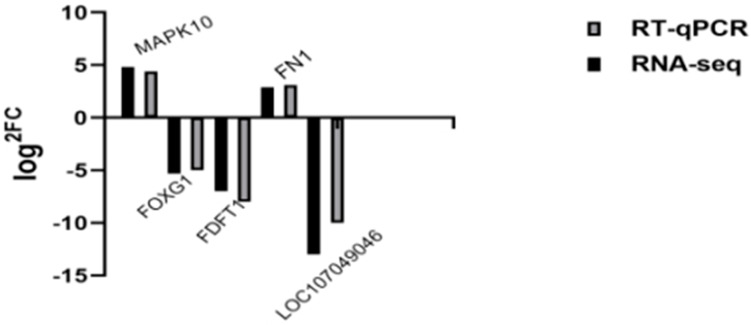
This is a validation diagram for a differentially expressed gene.

This study selected five differentially expressed genes for RT-qPCR validation (Figure 10). Results showed that MAPK10 was upregulated 4.8-fold, FOXG1 was downregulated 5.3-fold, FDFT1 was downregulated 7-fold, FN1 was upregulated 2.9-fold, and LOC107049046 was downregulated 12-fold. The log2FC values from transcriptome sequencing and qPCR were largely consistent, confirming the reliability of the transcriptome data.

## 4. Discussion

This study employed transcriptomic sequencing to profile gene expression changes in the kidneys, spleen, and liver of chickens under co-infection with ALV and *S. pullorum*, compared to single-infection and control groups. The results revealed distinct organ-specific transcriptional responses and identified key pathways and candidate genes potentially involved in the pathogenesis of co-infection.

Among the differentially enriched pathways, this study identified those significantly associated with cancer, tumorigenesis, and immune responses. In kidney tissues, genes differentially expressed in the co-infection group were enriched in pathways related to immune functions, such as the intestinal IgA production immune network [21], as well as metabolic processes including biosynthesis and metabolism of exogenous substances and redox enzyme activity. Notably, several ribosomal protein genes, including RPS3A [22] and RPL18A, showed downregulated expression. Literature indicates that some ribosomal proteins, such as RPL13A [23] and RPS3, have immunoregulatory roles [24,25]. In pathways involved in exogenous substance metabolism, significant changes were observed in cytochrome P450 genes like CYP2C23 and CYP1A, which are essential for renal metabolism [26]. In single-infection groups, the ALV-infected group mainly enriched pathways related to steroid and terpenoid biosynthesis, involving genes such as CYP51A1, DHCR24 [27], IDI2, and FDPS [28]. Conversely, the avian typhoid infection group primarily enriched the oxidative phosphorylation pathway [29].

In the spleen, genes differentially expressed in the co-infection group were enriched in functions related to the Notch signaling pathway, such as JAG1 downregulation [30], and regulation of endothelial cell proliferation, involving genes like IGF2 and NGFR [31,32]. In the ALV infection group, differentially expressed genes showed significant enrichment in calcium ion signaling pathways, ECM–receptor interactions, and MAPK signaling pathways, with altered expression of HRH1, CYSLTR2, and EDNRA [33]. The chicken white diarrhea infection group primarily exhibited enrichment in pathways including cell adhesion, focal adhesions, and ECM–receptor interactions, with genes such as COL1A1 [34] and MYL6 showing altered expression.

In the liver, differentially expressed genes from the co-infection group were extensively enriched across multiple metabolic pathways, including peroxisome function, amino acid and fatty acid metabolism, steroid biosynthesis, and PPAR signaling pathways [35]. Notably, genes like SQLE, MSMO1, FDFT1, and CYP51A1 displayed significant expression differences within the steroid synthesis pathway [36]. The enrichment patterns in single-infection groups showed similarities to those in the kidneys: the ALV-infected group was enriched in steroid hormone biosynthesis and PPAR signaling pathways, while the chicken typhoid-infected group was enriched in oxidative phosphorylation and respiratory electron transport chain pathways.

Furthermore, to clarify the specific effects of co-infection separate from single infections, we performed direct intergroup comparisons. Our analysis showed that in the kidneys, differentially expressed genes between the co-infection group (Group A) and the ALV-infected group (Group B) were significantly enriched in the PPAR signaling pathway. Notably, the rate-limiting enzyme genes ACOX1 [16] and CPT1A, which are involved in fatty acid oxidation within this pathway, showed downregulated expression. This indicates that co-infection and ALV monoinfection may regulate renal lipid metabolism through distinct molecular mechanisms. Detailed data are presented in Appendix E.

Across all three organs, eight candidate genes showed consistently significant expression changes in the co-infection group. These include upregulated SQLE, MSMO1, MAPK10, and LOC101747704, and downregulated FDFT1, FOXG1, LOC107049046, and FN1. Among these, MAPK10—a member of the JNK signaling pathway involved in apoptosis and inflammation—may play a role in co-infection-induced cellular stress responses. The unannotated genes LOC107049046 and LOC101747704, given their infection-specific and extreme expression patterns, warrant further investigation as potential novel regulators in avian co-infection. The remaining genes, particularly those involved in cholesterol metabolism (SQLE, MSMO1, FDFT1) and cell adhesion (FN1), provide promising directions for future functional studies.

In conclusion, this multi-organ transcriptomic analysis reveals that co-infection with ALV and S. Pullorum induces distinct molecular signatures not observed in single infections, with organ-specific pathway perturbations and a set of robust candidate genes. These findings provide a foundational framework for future studies aiming to dissect the mechanistic basis of co-infection pathogenesis and identify potential targets for intervention.

It should be noted that the sample size in this study (*n* = 3 per group) is relatively modest, which may constrain the statistical power and the extent to which the findings can be generalized. Although this sample size is commonly used in transcriptomic studies involving animal models due to practical and ethical considerations, future studies with larger cohorts are warranted to validate our key findings and further elucidate the biological mechanisms underlying ALV and S. Pullorum co-infection.

## 5. Conclusions

This study employed transcriptome sequencing technology to further investigate the molecular mechanisms underlying co-infection with ALV and *S. pullorum*, identifying multiple differentially expressed genes and pathways associated with immunity and tumourigenesis. Functional enrichment analysis revealed the crucial roles of pathways such as the PPAR signaling pathway and ECM–receptor interactions in the co-infection of ALV and *S. pullorum*. Candidate gene screening and real-time quantitative PCR validation further confirmed the potential functions of these genes. These findings provide a crucial molecular foundation for in-depth investigation into the regulatory mechanisms underlying the co-infection phenomenon of ALV and *S. pullorum*.

## Figures and Tables

**Table 1:** Principal reagents and consumables for the experiments.

Consumables Name	Manufacturer
Sterile PE gloves	Guangming, Shenzhen, China
Disposable Nitrile Gloves	Bioshar, Hefei, China
Face Mask	Biosharp, Hefei, China
RNA Preservation Solution	Solarbio, Beijing, China
Aluminum Foil	Clinlay, Shanghai, China
Surgical Scissors	Corning, Corning, NY, USA
PBS Buffer	Solarbio, Beijing, China
Blood Collection Tubes	Junuo, Taizhou, China
200 μL,1.5 mL Blood Collection Tubes	Axygen, Union City, CA, USA
10 μL, 200 μL, 1000 μL RNase-Free Pipette Tips	Biosharp, Hefei, China

**Table 2:** Principal Experimental Apparatus.

Instruments Name	Manufacturer
Bench-top High-speed Refrigerated Centrifuge	Eppendorf, Hamburg, Germany
Real-time Quantitative PCR System	Bio-Rad, Hercules, CA, USA
Thermostatic Magnetic Stirrer	Eppendorf, Hamburg, Germany
Ultra-low Temperature Freezer	Sanyo, Osaka, Japan
Full-wavelength Microplate Reader	Bio-Rad, Hercules, CA, USA
Pure Water Distillation System	Millipore, Saint Louis, MO, USA
Thermostatic Shaker Incubator	Binder, Neckarsulm, Germany
Personal Computer	Microsoft, Redmond, WA, USA
Nanodrop	Thermo Fisher Scientific, Waltham, MA, USA

**Table 3:** Transcriptome Primer Design Sequence.

Gene	Forward Primer (5′→3′)	Reverse Primer (5′→3′)
FDFT1	F: GAGGGGTGGTGAAGATTCGG	R: CAAGGGACAGCCAAGTCCAC
MAPK10	F: ACCATTGAGGAGTGGAAAGAACT	R: CACCTGAAGGAGATGGCTGT
FOXG1	F: CGATAGACTCGTCAACGGGG	R: GTTCACGGAGCAGGGGTTTA
FN1	F: TTGGAGAGCAGTGGCAGAAG	R: GGCAGTTGACGTTGGTGTTT
LOC107049046	F: GTGAGCAGGCAAAGGGGTTA	R: TTTACTCCCGAGCCATCAGC

**Figure A1:**
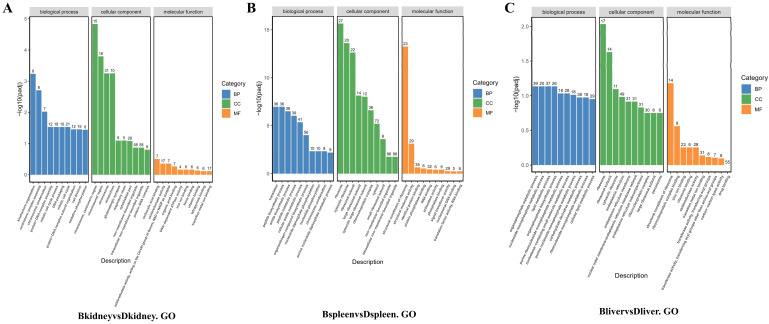
GO enrichment analysis of significantly differentially expressed genes in the avian leukosis virus-infected group, shown for the kidney (**A**), spleen (**B**), and liver (**C**), respectively.

**Figure A2:**
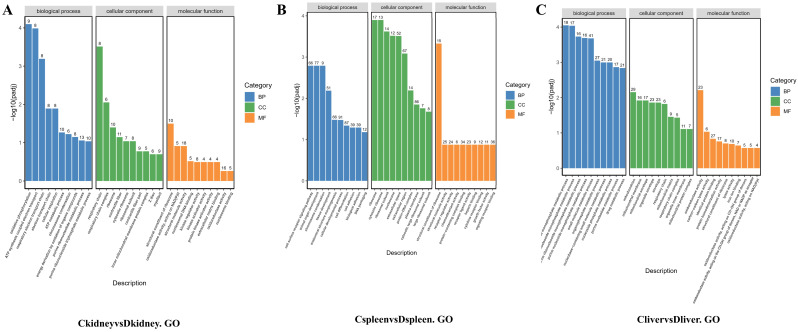
GO enrichment analysis of significantly differentially expressed genes in the kidney(**A**), spleen(**B**), and liver(**C**) of the *Salmonella pullorum*-infected group, respectively.

**Figure A3:**
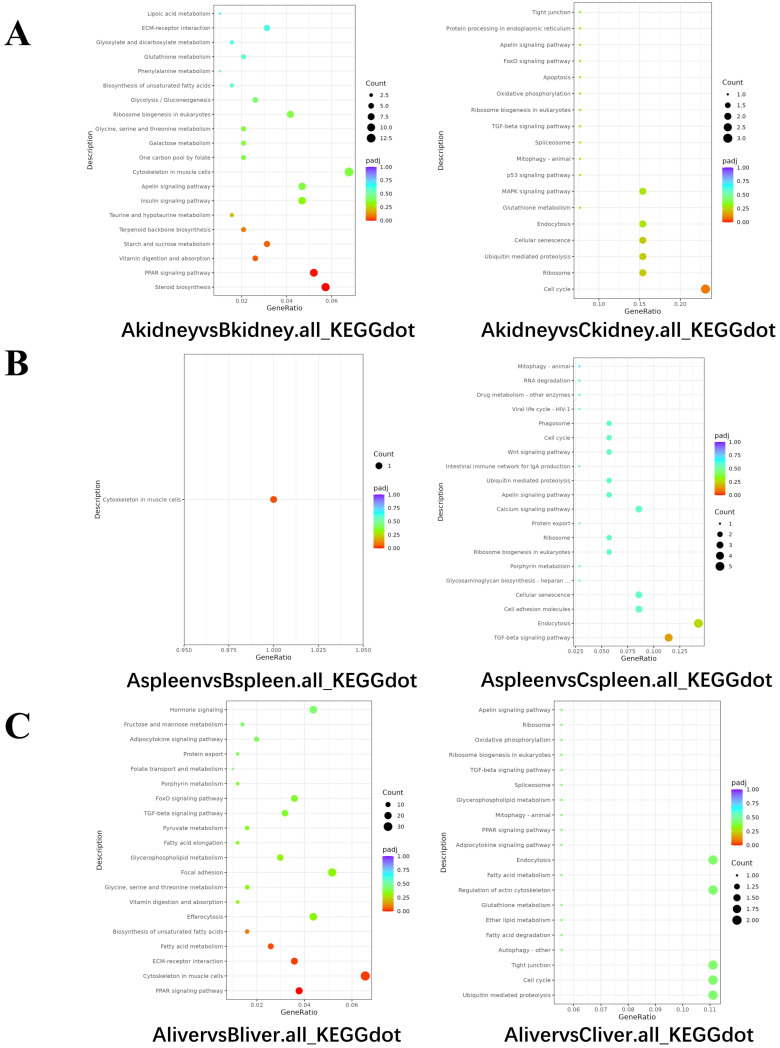
KEGG Pathway Diagram for the Co-Infection Group Compared with the Avian Leukosis Virus Infection Group and the Pullorum Disease Infection Group, Respectively. (**A**) Kidney; (**B**) Spleen; (**C**) Liver.

## Data Availability

The data presented in this study are openly available in the NCBI database under accession number PRJNA1247184.

## Appendix A. Sequencing Quality Summary

**Table A1:** Summary of sequencing data quality for 36 samples.

Sample	raw_reads	raw_bases	clean_reads	clean_bases	error_rate	Q20	Q30	GC_pct
NCliver1	47,122,838	7.07G	45,702,830	6.86G	0.01	99.07	96.25	49.74
NCspleen1	45,099,616	6.76G	43,751,982	6.56G	0.01	98.62	95.18	49
NCkidney1	43,523,442	6.53G	41,990,072	6.3G	0.01	98.93	95.93	48.47
NCliver2	45,149,892	6.77G	43,686,118	6.55G	0.01	99.09	96.38	48.25
NCspleen2	45,865,982	6.88G	44,546,546	6.68G	0.01	98.69	95.35	48.26
NCkidney2	44,313,696	6.65G	43,013,900	6.45G	0.01	98.93	95.93	49.08
NCliver3	44,521,762	6.68G	43,435,610	6.52G	0.01	98.99	96.15	49.07
NCspleen3	42,953,154	6.44G	41,824,592	6.27G	0.01	98.75	95.53	48.11
NCkidney3	43,971,384	6.6G	42,581,212	6.39G	0.01	98.9	95.84	48.56
Coliver1	46,571,970	6.99G	42,862,266	6.43G	0.01	98.53	96.12	50.03
Cospleen1	47,313,216	7.1G	44,073,998	6.61G	0.01	98.5	96.02	50.06
Cokidney1	46,780,012	7.02G	43,835,596	6.58G	0.01	98.48	96.01	48.76
Coliver2	46,386,192	6.96G	42,679,216	6.4G	0.01	98.37	95.75	49.7
Cospleen2	39,558,874	5.93G	35,798,054	5.37G	0.01	98.44	95.93	43.41
Cokidney2	48,383,918	7.26G	44,105,522	6.62G	0.01	98.69	96.55	49.31
Coliver3	45,346,502	6.8G	42,079,876	6.31G	0.01	98.64	96.36	49.34
Cospleen3	53,856,744	8.08G	48,696,534	7.3G	0.01	98.61	96.34	49.69
Cokidney3	43,133,104	6.47G	38,818,760	5.82G	0.01	98.65	96.35	49.78
ALVliver1	47,082,034	7.06G	42,710,160	6.41G	0.01	97.99	94.77	47.81
AlVspleen1	46,604,880	6.99G	45,630,280	6.84G	0.03	97.71	94.01	50.61
ALVkidney1	43,504,608	6.53G	43,273,194	6.49G	0.01	97.61	94.03	48.29
ALVliver2	46,063,920	6.91G	45,761,888	6.86G	0.01	97.78	94.43	47.9
AlVspleen2	48,325,626	7.25G	48,056,206	7.21G	0.01	98.68	96.54	49.77
ALVkidney2	47,124,032	7.07G	45,503,784	6.83G	0.01	97.82	94.49	48.41
ALVliver3	46,886,300	7.03G	42,780,776	6.42G	0.01	97.94	94.74	46.88
AlVspleen3	47,763,944	7.16G	47,493,918	7.12G	0.01	98.65	96.49	49.48
ALVkidney3	49,016,288	7.35G	47,608,468	7.14G	0.01	97.77	94.32	48.74
SPliver1	49,309,940	7.4G	45,788,322	6.87G	0.01	98.49	95.95	49.04
SPspleen1	56,965,314	8.54G	55,605,910	8.34G	0.03	97.23	93.09	51.37
SPkidney1	46,791,238	7.02G	42,784,064	6.42G	0.01	98.61	96.27	50.15
SPliver2	48,055,566	7.21G	47,486,342	7.12G	0.01	98.17	95.44	52.58
SPspleen2	49,245,736	7.39G	48,085,522	7.21G	0.03	97.08	92.9	50.99
SPkidney2	47,284,290	7.09G	42,577,464	6.39G	0.01	98.42	95.91	46.53
SPliver3	47,099,914	7.06G	42,746,482	6.41G	0.01	98.46	96.02	50.9
SPspleen3	47,335,950	7.1G	44,007,364	6.6G	0.01	98.45	95.91	50.93
SPkidney3	49,532,920	7.43G	46,118,112	6.92G	0.01	98.42	95.92	49.14

## Appendix B. Statistics Table Showing the Alignment of Reads to the Reference Genome

**Table A2:** Statistics of reads alignment to the reference genome for 36 samples.

Sample	total_reads	total_map	unique_map	multi_map	read1_map	read2_map
NCliver1	45,702,830	42,369,669 (92.71%)	40,856,549 (89.4%)	1,513,120 (3.31%)	20,492,174 (44.84%)	20,364,375 (44.56%)
NCspleen1	43,751,982	37,888,197 (86.6%)	36,735,018 (83.96%)	1,153,179 (2.64%)	18,445,875 (42.16%)	18,289,143 (41.8%)
NCkidney1	41,990,072	38,429,474 (91.52%)	37,543,778 (89.41%)	885,696 (2.11%)	18,838,369 (44.86%)	18,705,409 (44.55%)
NCliver2	43,686,118	40,964,593 (93.77%)	39,713,532 (90.91%)	1,251,061 (2.86%)	19,913,920 (45.58%)	19,799,612 (45.32%)
NCspleen2	44,546,546	39,183,048 (87.96%)	38,095,011 (85.52%)	1,088,037 (2.44%)	19,127,876 (42.94%)	18,967,135 (42.58%)
NCkidney2	43,013,900	39,142,310 (91.0%)	38,197,259 (88.8%)	945,051 (2.2%)	19,174,461 (44.58%)	19,022,798 (44.22%)
NCliver3	43,435,610	39,792,800 (91.61%)	38,439,477 (88.5%)	1,353,323 (3.12%)	19,284,490 (44.4%)	19,154,987 (44.1%)
NCspleen3	41,824,592	36,988,325 (88.44%)	35,956,252 (85.97%)	1,032,073 (2.47%)	18,053,860 (43.17%)	17,902,392 (42.8%)
NCkidney3	42,581,212	38,804,192 (91.13%)	37,884,522 (88.97%)	919,670 (2.16%)	19,013,484 (44.65%)	18,871,038 (44.32%)
Coliver1	42,862,266	38,763,208 (90.44%)	36,185,439 (84.42%)	2,577,769 (6.01%)	18,172,977 (42.4%)	18,012,462 (42.02%)
Cospleen1	44,073,998	39,696,349 (90.07%)	37,436,384 (84.94%)	2,259,965 (5.13%)	18,809,145 (42.68%)	18,627,239 (42.26%)
Cokidney1	43,835,596	40,597,072 (92.61%)	37,176,493 (84.81%)	3,420,579 (7.8%)	18,685,423 (42.63%)	18,491,070 (42.18%)
Coliver2	42,679,216	39,033,426 (91.46%)	37,583,939 (88.06%)	1,449,487 (3.4%)	18,905,843 (44.3%)	18,678,096 (43.76%)
Cospleen2	35,798,054	32,976,692 (92.12%)	31,474,788 (87.92%)	1,501,904 (4.2%)	15,794,575 (44.12%)	15,680,213(43.8%)
Cokidney2	44,105,522	39,800,126 (90.24%)	38,557,254 (87.42%)	1,242,872 (2.82%)	19,361,139 (43.9%)	19,196,115 (43.52%)
Coliver3	42,079,876	38,574,311 (91.67%)	36,374,680 (86.44%)	2,199,631 (5.23%)	18,280,484 (43.44%)	18,094,196 (43.0%)
Cospleen3	48,696,534	43,174,258 (88.66%)	41,867,891 (85.98%)	1,306,367 (2.68%)	21,029,074 (43.18%)	20,838,817 (42.79%)
Cokidney3	38,818,760	34,999,116 (90.16%)	33,457,142 (86.19%)	1,541,974 (3.97%)	16817425 (43.32%)	16,639,717 (42.87%)
ALVliver1	42,710,160	38,331,689 (89.75%)	36,364,508 (85.14%)	1,967,181 (4.61%)	18,209,389 (42.63%)	18,155,119 (42.51%)
AlVspleen1	45,630,280	41,072,961 (90.01%)	39,790,131 (87.2%)	1,282,830 (2.81%)	19,959,354 (43.74%)	19,830,777 (43.46%)
ALVkidney1	43,273,194	37,626,261 (86.95%)	35,111,071 (81.14%)	2,515,190 (5.81%)	17,652,033 (40.79%)	17,459,038 (40.35%)
ALVliver2	45,761,888	40,335,525 (88.14%)	37,935,714 (82.9%)	2,399,811 (5.24%)	19,018,811 (41.56%)	18,916,903 (41.34%)
AlVspleen2	48,056,206	42,958,995 (89.39%)	41,815,452 (87.01%)	1,143,543 (2.38%)	20,943,556 (43.58%)	20,871,896 (43.43%)
ALVkidney2	45,503,784	39,719,422 (87.29%)	38,104,267 (83.74%)	1,615,155 (3.55%)	19,159,341 (42.1%)	18,944,926 (41.63%)
ALVliver3	42,780,776	38,186,042 (89.26%)	36,426,385 (85.15%)	1,759,657 (4.11%)	18,237,858 (42.63%)	18,188,527 (42.52%)
AlVspleen3	47,493,918	43,301,247 (91.17%)	40,764,003 (85.83%)	2,537,244 (5.34%)	20,406,461 (42.97%)	20,357,542 (42.86%)
ALVkidney3	47,608,468	41,175,348 (86.49%)	39,385,541 (82.73%)	1,789,807(3.76%)	19,783,452 (41.55%)	19,602,089 (41.17%)
SPliver1	45,788,322	43,083,740 (94.09%)	40,383,319 (88.2%)	2,700,421 (5.9%)	20,293,096 (44.32%)	20,090,223 (43.88%)
SPspleen1	55,605,910	49,682,454 (89.35%)	47,716,412 (85.81%)	1,966,042 (3.54%)	23,975,454 (43.12%)	23,740,958 (42.7%)
SPkidney1	42,784,064	38,329,865 (89.59%)	36,618,704 (85.59%)	1,711,161 (4.0%)	18,398,935 (43.0%)	18,219,769 (42.59%)
SPliver2	47,486,342	41,847,825 (88.13%)	38,537,451 (81.15%)	3,310,374 (6.97%)	19,362,546 (40.77%)	19,174,905 (40.38%)
SPspleen2	48,085,522	42,902,808 (89.22%)	41,147,678 (85.57%)	1,755,130 (3.65%)	20,675,946 (43.0%)	20,471,732 (42.57%)
SPkidney2	42,577,464	39,399,588 (92.54%)	39,028,482 (91.66%)	371,106 (0.87%)	19,581,145 (45.99%)	19,447,337 (45.68%)
SPliver3	42,746,482	37,661,195 (88.1%)	35,316,294 (82.62%)	2,344,901 (5.49%)	17,756,083 (41.54%)	17,560,211 (41.08%)
SPspleen3	44,007,364	39,407,403 (89.55%)	36,748,025 (83.5%)	2,659,378 (6.04%)	18,476,637 (41.99%)	18,271,388 (41.52%)
SPkidney3	46,118,112	42,972,879 (93.18%)	40,123,808 (87.0%)	2,849,071 (6.18%)	20,158,553 (43.71%)	19,965,255 (43.29%)
